# A newly isolated roseophage represents a distinct member of *Siphoviridae* family

**DOI:** 10.1186/s12985-019-1241-6

**Published:** 2019-11-06

**Authors:** Lanlan Cai, Ruijie Ma, Hong Chen, Yunlan Yang, Nianzhi Jiao, Rui Zhang

**Affiliations:** State Key Laboratory of Marine Environmental Science, College of Ocean and Earth Sciences, Institute of Marine Microbes and Ecospheres, Xiamen University, Xiamen, 361102 People’s Republic of China

**Keywords:** *Dinoroseobacter*, Roseophage, *Siphoviridae*, Genome sequence

## Abstract

**Background:**

Members of the *Roseobacter* lineage are a major group of marine heterotrophic bacteria because of their wide distribution, versatile lifestyles and important biogeochemical roles. Bacteriophages, the most abundant biological entities in the ocean, play important roles in shaping their hosts’ population structures and mediating genetic exchange between hosts. However, our knowledge of roseophages (bacteriophages that infect *Roseobacter*) is far behind that of their host counterparts, partly reflecting the need to isolate and analyze the phages associated with this ecologically important bacterial clade.

**Methods:**

vB_DshS-R4C (R4C), a novel virulent roseophage that infects *Dinoroseobacter shibae* DFL12^T^, was isolated with the double-layer agar method. The phage morphology was visualized with transmission electron microscopy. We characterized R4C in-depth with a genomic analysis and investigated the distribution of the R4C genome in different environments with a metagenomic recruitment analysis.

**Results:**

The double-stranded DNA genome of R4C consists of 36,291 bp with a high GC content of 66.75%. It has 49 genes with low DNA and protein homologies to those of other known phages. Morphological and phylogenetic analyses suggested that R4C is a novel member of the family *Siphoviridae* and is most closely related to phages in the genus *Cronusvirus*. However, unlike the *Cronusvirus* phages, R4C encodes an integrase, implying its ability to establish a lysogenic life cycle. A terminal analysis shows that, like that of λ phage, the R4C genome utilize the ‘cohesive ends’ DNA-packaging mechanism. Significantly, homologues of the R4C genes are more prevalent in coastal areas than in the open ocean.

**Conclusions:**

Information about this newly discovered phage extends our understanding of bacteriophage diversity, evolution, and their roles in different environments.

## Background

Bacteriophages or ‘phages’ are abundant and play important roles in shaping microbial population structures, mediating genetic exchange, and modulating biogeochemical cycling in the ocean [1, 2]. With rapid technological advances in DNA sequencing, culture-independent viral metagenomic studies have revealed that marine viruses carry extremely high, but largely uncharacterized genetic diversity [3, 4]. The large amount of unknown sequences is in great part due to the paucity of viral reference genome in the database. As an irreplaceable technique, the isolation and genomic analysis of new viruses could significantly contribute to the interpretation of overwhelming unknown sequences in the viromes [5, 6]. In addition, novel characterized phages can also provide valuable information on the biological features of viruses (such as morphology, infectious cycle, and host specificity) and extend our understanding of genome evolution, phage–host interactions, and phage ecology.

The *Roseobacter* lineage represents a major clade of marine heterotrophic bacteria, with versatile metabolic features, high genomic plasticity, and important biogeochemical roles [7–9]. The bacteria in this clade are globally distributed throughout the surface oceans, and have emerged as an important model organism for the study of marine microbial ecology [9]. Interestingly, many *Roseobacter* genomes contain intact prophages and nearly all harbor a conserved gene transfer agent (GTA) operon [10, 11], suggesting that they interact closely with phages. However, only a handful of roseophages have been isolated and characterized. Recently, Zhan et al. provided an up-to-date overview of the roseophages isolated from different lineages of *Roseobacter*, demonstrating the phylogenetic diversity of the roseophages and their multiple mutual effects on *Roseobacter* [12]. Therefore, the roseophage–*Roseobacter* could offer an ideal system to gain new insights into the diversity and evolution of phages and the relationships between phages and their bacterial hosts.

*Dinoroseobacter shibae* DFL12^T^ is one of the most prominent and well-studied members of the *Roseobacter* clade [13]. It has interesting and important metabolic traits, such as the ability to grow anaerobically and the adaption to dark-light cycles which allows the additional energy generation from light under heterotrophic and starvation conditions [14]. So far, four phages that infect *D. shibae* DFL12^T^ have been reported, three of which have a highly conserved genomic organization and belong to the N4-like genus of the family *Podoviridae* [15–17]. Only one *D. shibae* siphophage, which was isolated from an oligotrophic environment, has been sequenced and showed little similarity to known phages [18].

In this study, we report the isolation and characterization of another novel siphophage, vB_DshS-R4C, infecting *D. shibae* DFL12^T^. Microbiological and genomic analyses provide an overview of its features and its evolutionary relationships with other previously characterized phages. We demonstrate that R4C is a distinct member of the family *Siphoviridae*.

## Methods

### Phage isolation and purification

The host strain *D. shibae* DFL12^T^ was incubated in rich organic (RO) medium (1 M yeast extract, 1 M peptone, 1 M sodium acetate, artificial seawater, pH 7.5) at 37 °C with shaking at 180 rpm/min. The samples for virus isolation were collected from the coastal seawater of Xiamen, China, and filtered through a 0.2 μm membrane. To improve the chance of successful phage isolation, the viruses in the seawater were concentrated with tangential flow filtration through a 30-kDa cartridge (Millipore, CA, USA) and then mixed with *D. shibae* DFL12^T^ using double-layer agar method [18]. After overnight incubation at 37 °C, individual clear lytic plaques were picked, suspended in 1 mL of SM buffer (50 mM Tris-HCl [pH 7.5], 0.1 M NaCl, 8 mM MgSO_4_), and purified by replating at least five times to obtain a pure phage culture. The purified plaques were then eluted with SM buffer and stored at 4 °C for further usage.

### Host range

The lytic host range of the phage was determined by spotting dilutions onto lawns of 19 bacterial test strains, mainly from the genera *Roseobacter*, *Erythrobacter*, *Citromicrobium*, *Roseomonas*, and *Silicibacter*, as shown in Additional file 1: Table S1 [19]. The bacterial cultures (1 mL) in the exponential growth phase were added to 3 mL of molten RO agar medium (0.5% w/v agar). The mixture was then poured onto a solid agar plate (1.5% w/v agar), which was placed at room temperature (approximately 25 °C) to solidify. Diluted phage lysate (10 μL) was spotted onto the surface of each plate, incubated overnight at 37 °C, and then checked for the presence of lytic plaques.

### Lipid test

To investigate the presence of lipid in R4C, the phages were incubated with 0.2, 2%, or 20% (v/v) chloroform with vibration for 1 min and then kept at room temperature for 30 min. The titers of the phage were then determined by dropping it onto *D. shibae* DFL12^T^ plate to examine its sensitivity to chloroform.

### One-step growth curve

One-step growth curve was constructed to analyze the life cycle of R4C [20]. Briefly, the phage was added to 1 mL of log-phase *D. shibae* DFL12^T^ at a multiplicity of infection of 0.01, and then incubated for 25 min at room temperature in the dark. The unabsorbed phage particles were removed by centrifugation at 10,000×g for 5 min. After resuspended in 50 mL of RO medium, the suspension was incubated at 37 °C with continuous shaking. Samples were collected every 30 min and viral abundance was quantified with a double-agar plaque assay.

### Preparation of high-titer phage suspensions

High-titer phage suspensions for morphological observation and DNA extraction were prepared with cesium chloride (CsCl) gradient ultracentrifugation. Briefly, the phage was propagated in strain DFL12^T^ and collected after complete bacterial lysis. The culture was centrifuged at 10,000×g for 10 min and filtered through a 0.2 μm membrane. The phage suspension was precipitated with 1 M NaCl and polyethylene glycol (PEG) 8000 (10% w/v) overnight at 4 °C. The phage particles from the PEG pellet were purified with CsCl (1.5 g/mL in SM buffer) gradient centrifugation (200,000×g, 4 °C, 24 h). The phage bands were collected and dialyzed against SM buffer at 4 °C.

### Transmission electron microscopy (TEM)

The phage morphology was investigated with TEM. In brief, 10 μL of high-titer phage concentrate was placed on formvar, carbon-coated copper electron microscopy grids (200 mesh) and allowed to adsorb for 20 min. The phage particles were negatively stained with 1% (w/v) phosphotungstic acid for 1 min. Excess stain was removed with filter paper and the grids were air dried before examination with a JEM-2100 electron microscope (accelerating voltage of 120 kV).

### DNA extraction

For DNA extraction, the high-titer phage concentrate was treated with DNase I and RNase A at room temperature for 1 h to reduce host DNA contamination and then the DNase was inactivated at 65 °C for 15 min. The phage was lysed with proteinase K (50 μM), EDTA (20 mM), and sodium dodecyl sulfate (0.5% w/v) at 55 °C for 3 h. The phage DNA was extracted with the phenol/chloroform/isoamyl alcohol method and precipitated with ethanol. After quality and quantity checks with NanoDrop 2000 spectrophotometer and agarose gel electrophoresis, the genomic DNA was stored at − 80 °C until sequencing.

### Genome sequencing and analysis

The genomic DNA was sequenced on the Illumina HiSeq 2500 platform with pair-end (PE) read sizes of 100 bp. The raw reads were quality checked with FastQC and trimmed with FASTX-Toolkit. On average, Illumina PE reads 1 and reads 2 had > 90% and > 75% of bases with a quality score of at least 30 (Q30), respectively. The sequences were assembled with the Velvet software (v1.2.03) [21]. The phage termini and DNA-packing strategy were predicted with PhageTerm [22], with a mapping coverage setting of 20,000. The GeneMarkS online server and RAST (http://rast.nmpdr.org/) were used to identify putative open reading frames (ORFs), and the results were merged and checked manually. Gene annotation was performed with the algorithms of a BLAST search (National Center for Biotechnology Information, NCBI) against the nonredundant (nr) nucleotide database, with e-values of < 10^− 5^. The presence of tRNAs was examined with tRNAscan-SE. Comparison of genomes between R4C and other related phages were performed using BLAST. The complete genome sequence was submitted to the GenBank database under accession number MK882925.

### Phylogenetic analyses

In this study, the major capsid protein, large terminase subunit (TerL), and GTA-like sequences of R4C were used to construct phylogenetic trees to analyze its evolutionary relationships. Homologues were identified with BLASTP against the NCBI nr database using the acid-amino sequences as queries. Multiple sequence alignments were constructed with ClustalW, with the default parameters. Phylogenetic trees were constructed with the maximum likelihood method, with 1000 bootstrap replicates, in the MEGA 6.0 software (http://www.megasoftware.net/). The accession numbers of the viruses used in the alignments and phylogenetic analyses are listed on the trees.

### Recruitment of metagenomic data

To analyze the distribution of the R4C genome in different environments, homologues of the R4C ORFs were recruited from the Global Ocean Sampling (GOS) metagenomes and Pacific Ocean Virome (POV). The reads was recruited with tBLASTn using a threshold e-value of 10^− 5^, a bit score of > 40, and a minimum amino-acid length of 30, as previously described [23].

## Results and discussion

### Biological characterization of R4C

**Fig. 1:**
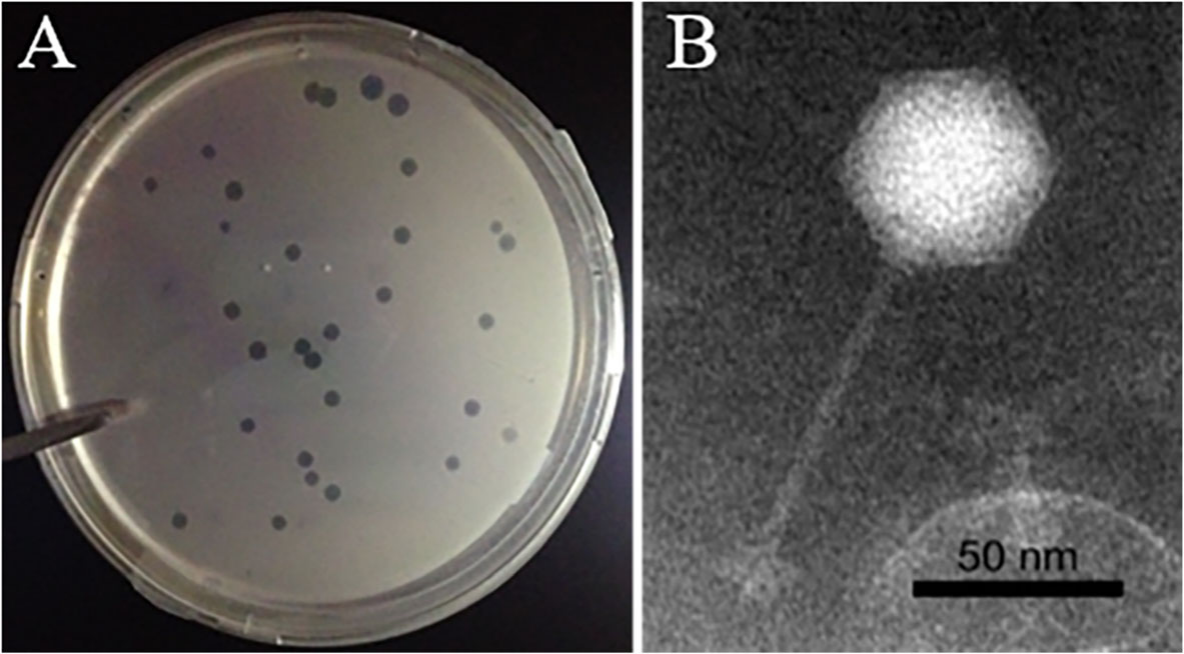
Plaques (**a**) and transmission electron microscopic image (**b**) of roseophage vB_DshS-R4C

In this study, a novel phage, designated vB_DshS-R4C, was isolated from the coastal seawater of Xiamen, China (24.45°N, 118.08°E) using the double-layer agar method. Most roseophages have been isolated from coastal waters, except one roseosiphophage, which was isolated from the oligotrophic South China Sea [18]. R4C formed clear plaques, with sizes ranging from 1.5 to 2.0 mm in diameter, and well-defined boundaries on the *D. shibae* DFL12^T^ bacterial host strain (Fig. 1a).

A TEM analysis revealed that R4C has an isometric and icosahedral head, with an estimated diameter of 55 ± 2 nm. The phage has a long noncontractile tail, measuring 82 ± 3 nm (Fig. 1b). According to its morphological characteristics and the guidelines of the International Committee on the Taxonomy of Viruses, phage R4C belongs to the family *Siphoviridae* in the order *Caudovirales* (tailed phages). Until now, over 96% of the phages reported in the scientific literature belong to the order *Caudovirales*, and the siphoviruses comprise approximately 61% of the tailed phages [24]. However, only 33% of the known roseophages belong to *Siphoviridae*, and the rest to the families *Podoviridae* and *Microviridae* [12].

The host range of this newly isolated phage was assayed with the spot test. Among all the 19 strains tested, phage R4C can only infect *D. shibae* DFL12 (Additional file 1: Table S1), but other yet-to-be discovered hosts cannot be ruled out here. This result is consistent with the previous finding that roseophages seem to have narrow host ranges [12]. The suspensions of R4C treated with three different concentrations of chloroform showed obvious lytic plaques, indicating the absence of lipids outside the capsid, which is commonly observed in phages of the order *Caudovirales* [18].

**Fig. 2:**
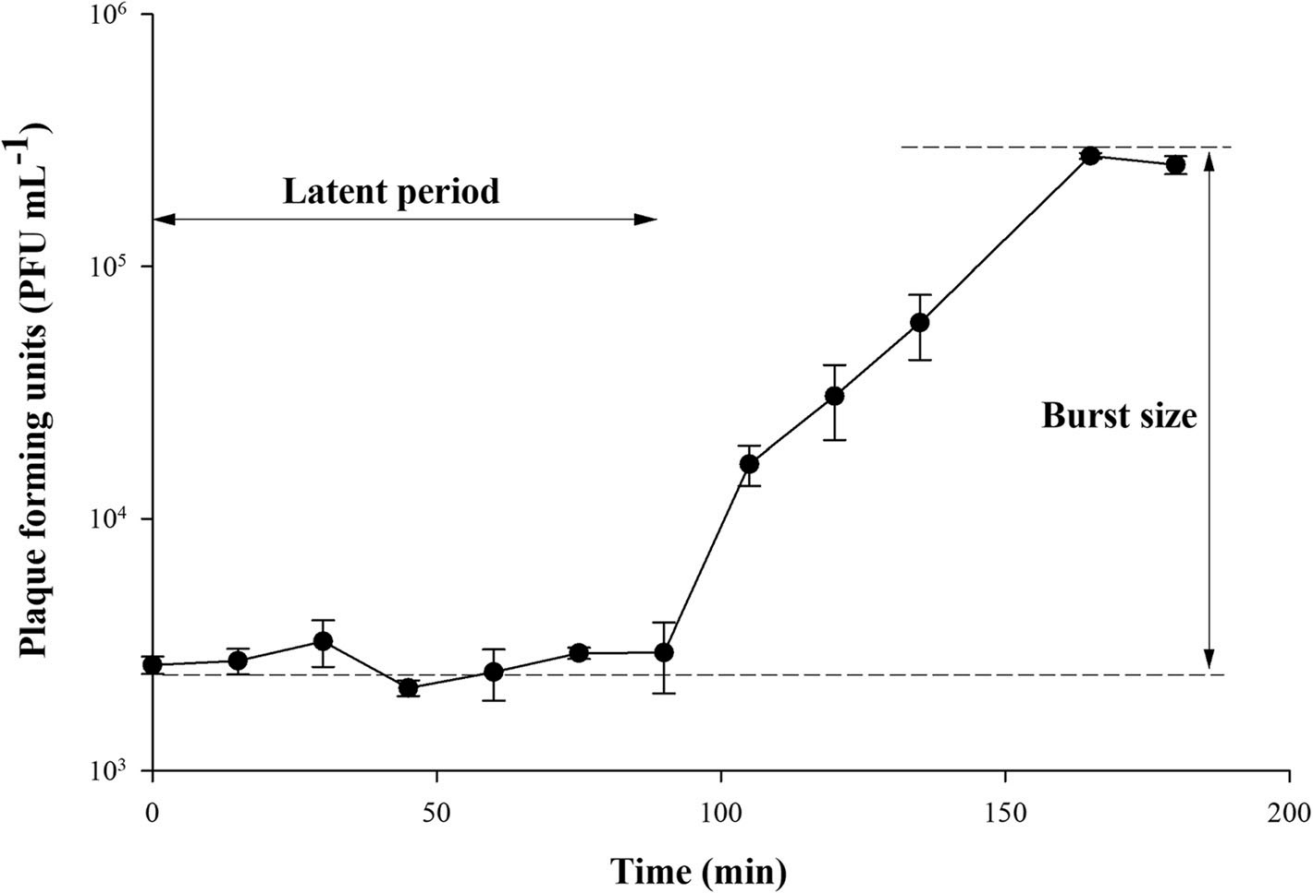
One-step growth curve of roseophage vB_DshS-R4C

To further understand the lytic cycle of R4C, a one-step growth curve was constructed, which showed a latent period of about 90 min for R4C (Fig. 2). The latent period is defined as the period between phage adsorption and the beginning of the burst, before any significant increase in phage particles. R4C showed a small burst size of 96 plaque-forming units (PFU)/cell, calculated as the ratio of the final number of phage particles at the growth plateau (2.5 h, as shown in Fig. 2) to the initial number of infected bacterial cells at the beginning of the latent period. The burst size of R4C is a bit larger than that of R5C, the other siphophage infecting *D. shibae* DFL12 (65 PFU cell^− 1^), and falls into the broad values of roseophages, ranging from 10 to 1500 cell^− 1^ [18].

### Bioinformatic analysis of the genomic sequence

#### General genomic features

Genome assembly based on 3,048,949 PE reads yielded a single contig with an average coverage of 19,731×. The genome of R4C is a double-stranded DNA (dsDNA) molecule consisting of 36,291 bp, with a high G + C content of 66.75%, which is very similar to the average G + C content (66.02%) of its host. The average genome size of the phages within the family *Siphoviridae* is estimated to be 53.70 kb [25]. Therefore, R4C has a relatively small genome within this family, reflecting the more retrenching virion structure. The properties of the genome, such as the positions, directions, and putative functions of each gene, are summarized in Additional file 1: Table S2. In total, 49 putative ORFs were predicted in the R4C genome, with 48 ORFs on the positive strand and one ORF on the negative strand. A total of 35,145 nucleotides (96.59% of the genome) are involved in coding putative proteins. The average gene length is 715 bp, with a range of 111 to 4344 nucleotides. Only 22 predicted ORFs (44.90%) were predicted to be functional, whereas 27 were assigned to hypothetical proteins. No tRNA sequences were detected in the R4C genome with the tRNAscan-SE program, indicating that the phage is completely reliant on the host tRNA for its protein synthesis. Genome annotation with BLASTP identified different functional clusters, including those involved in DNA packaging, virion morphogenesis, DNA manipulation, and regulation.

### Phage DNA-packaging mechanism

A termini analysis that can detect the DNA-packaging mechanisms of dsDNA phages was implemented using the PhageTerm software. Toward the end of the infection cycle, dsDNA phages generally form concatemeric DNA, which is cleaved by terminase and then encapsulated in a preformed empty prohead. Although there are several different phage DNA-packaging mechanisms, two modes are well characterized: the cohesive ends (*cos*) and headful (*pac*) packaging types. For phages with DNA cohesive ends, such as the λ-like phages, terminase recognizes the *cos* site and introduces a staggered cut, generating a unit-length encapsidated genome. By comparison, in the headful packaging phages (such as the T4, P22, or P1 phages), packaging starts by cleavage at a *pac* site and ends when the procapsid reaches its capacity. These phages encapsidate more than one unit-length of the phage genome (typically 102–110%), producing a virion DNA with a terminally redundant sequence. Analysis of the phage R4C genome identified a 14-bp 5′ protruding cohesive end region, upstream from the terminase small subunit gene, suggesting that the R4C genome utilizes the cohesive ends packaging strategy of the λ-like phages. The large terminase subunit gene is often conserved amongst the tailed bacteriophages that use either the *cos-* or *pac*-type packaging mechanisms. A phylogenetic analysis of R4C TerL, together with those of phages with known packaging mechanisms, also clustered R4C into the clade of phages that utilize λ-like DNA packaging (see below).

### GTA cluster

**Fig. 3:**
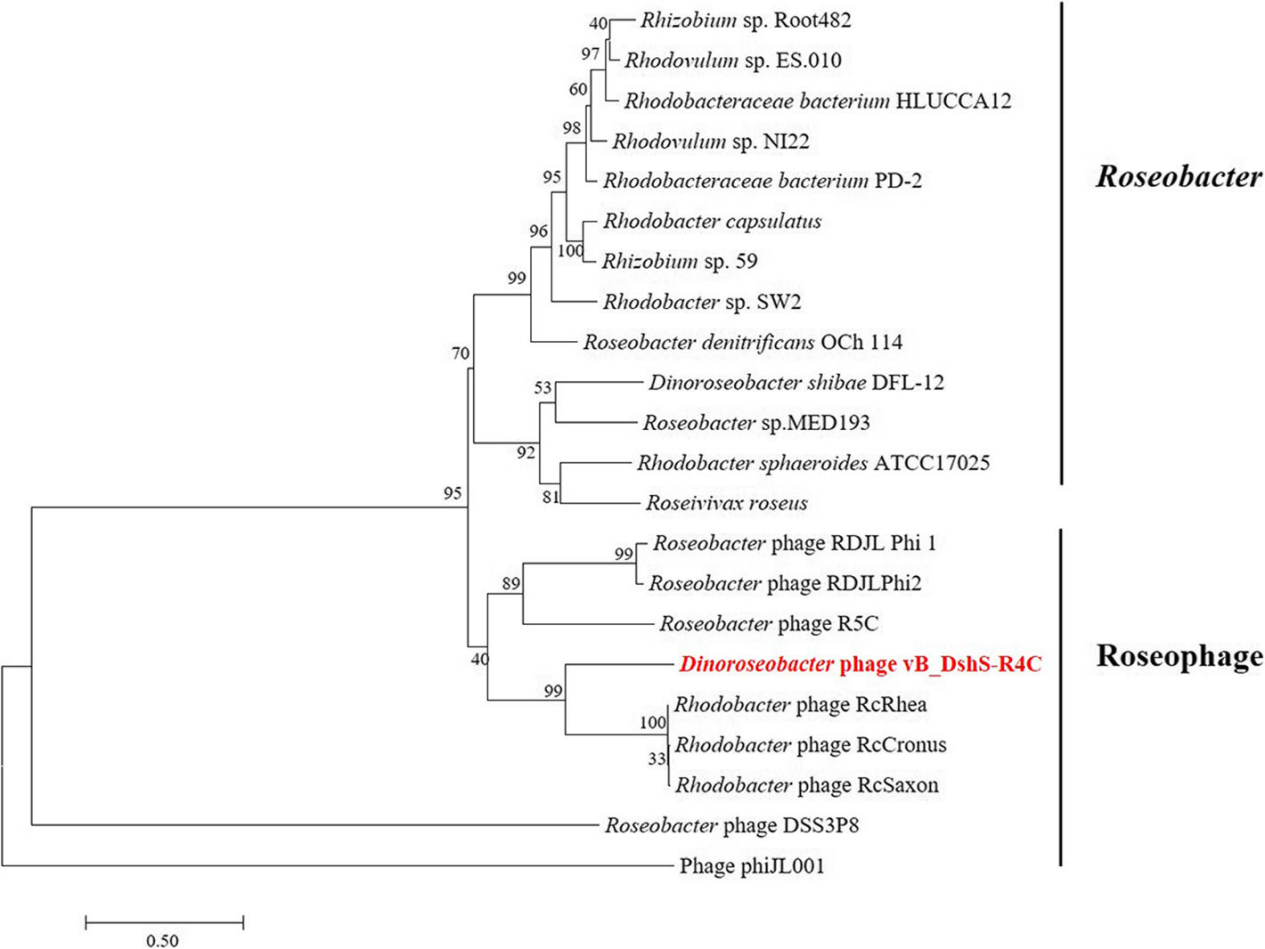
Maximum likelihood phylogenetic tree of GTA-like genes from *Roseobacter* and Roseophages. Maximum likelihood analyses with 1000 bootstrap replicates were used to derive the trees based on amino-acid sequences. Bootstrap values are shown above the major nodes. Phage vB_DshS-R4C is marked in red. Scale bars represent 0.5 fixed mutations per amino-acid position

GTA, a phage-like entity that encodes 15 to 17 genes [26], is a well-preserved genetic module found in the *Roseobacter* lineage. Four GTA-like genes were identified by BLASTP in the R4C genome, downstream from the DNA-packaging and structural genes, such as those encoding the head-to-tail joining protein, major capsid protein, and tail tape measure protein. ORF 13 was identified as a glycoside hydrolase, and is homologous to gene 12 of the GTA operon found in *Rhodobacter capsulatus* (RcGTA). ORF 14 is most closely related to gene 13 of RcGTA. ORF 15 contains a phage-related cell wall peptidase domain that may help the phage penetrate the bacterial cell wall. ORF 16 is the largest gene in the RcGTA operon and known to be involved in host specificity. These four hallmark GTA-like genes have also been found in other roseophages within the family *Siphoviridae* [12]. In the phylogenetic analysis based on these four GTA-like sequences, the roseophages formed distinct clades from their hosts, indicating the independent evolution of the phage-encoded and host GTA-like genes (Fig. 3).

### Comparative genomic analysis

**Fig. 4:**
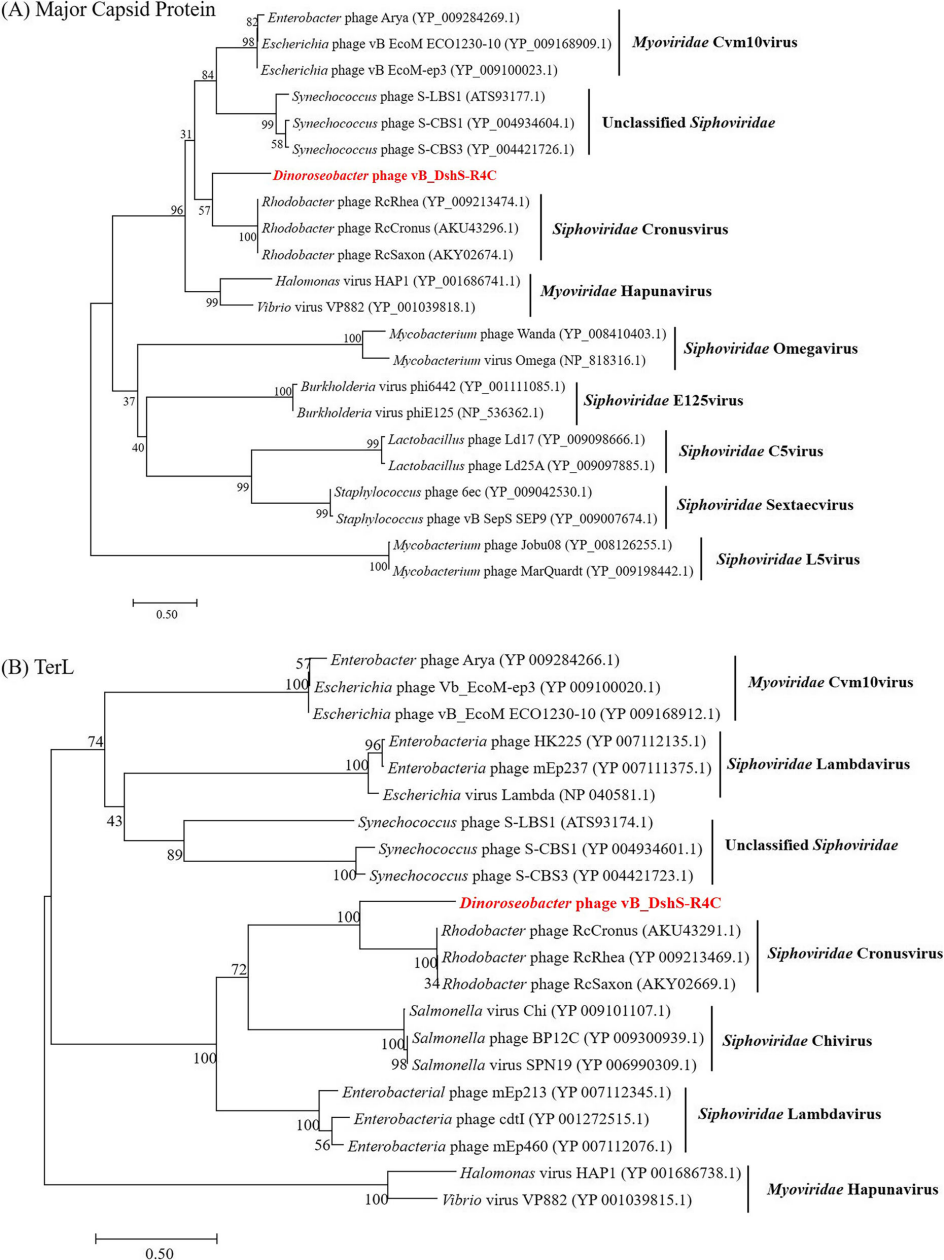
Phylogenetic trees of major capsid protein (**a**) and TerL (**b**) of R4C and other known phages. Maximum likelihood analyses with 1000 bootstrap replicates were used to derive the trees based on amino-acid sequences. Bootstrap values are shown above the major nodes. Phage vB_DshS-R4C is marked in red. Numbers in brackets represent the corresponding GenBank IDs. Scale bars represent 0.5 fixed mutations per amino-acid position

**Fig. 5:**
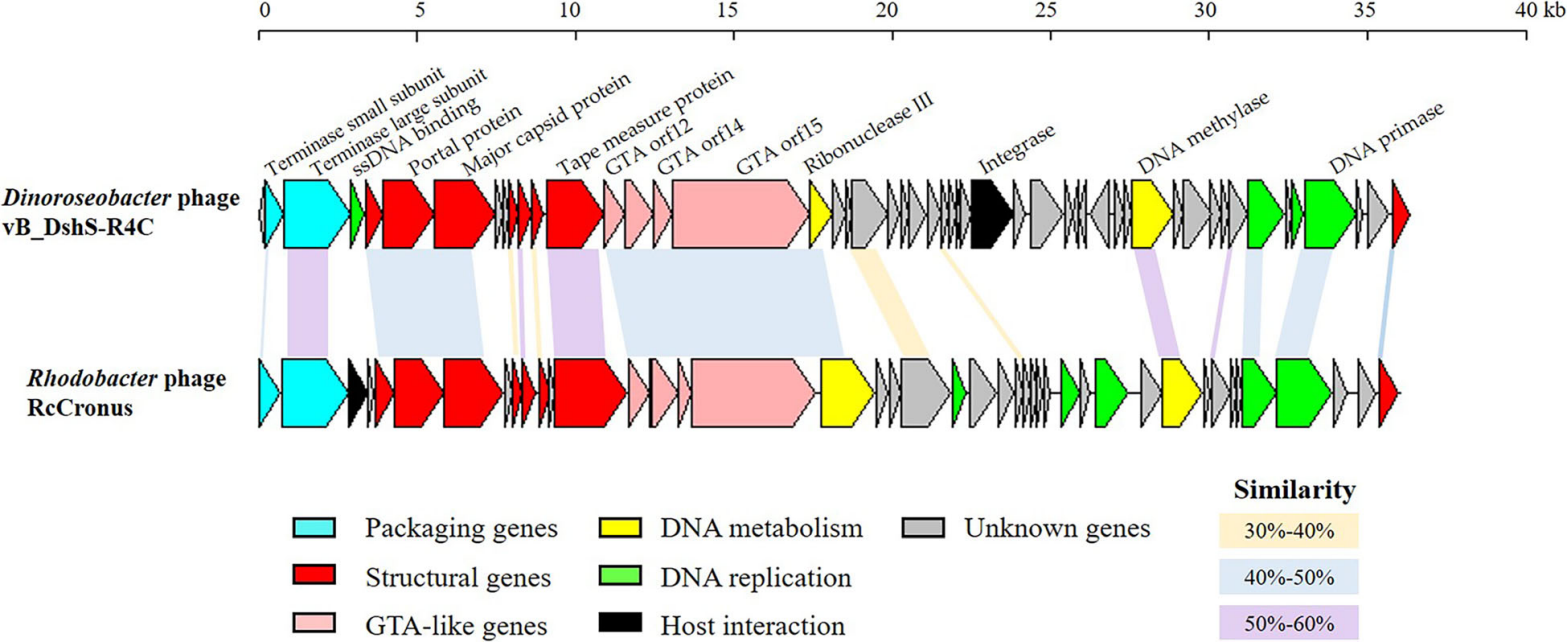
Comparison of the vB_DshS-R4C and RcCronus genomes. ORFs are indicated by leftward- or rightward-oriented arrows according to the direction of transcription. Colored arrows denote putative functions assigned according to BLASTP similarity. Homologous ORFs are connected by shadowing. Colors of shadows correspond to different levels of amino-acid identity

The International Committee on the Taxonomy of Viruses (ICTV) has taken a holistic approach to the classification of phages, using the overall DNA and protein sequence identities, coupled to phylogenetic analyses. An initial BLASTN analysis of the complete genome sequence of R4C revealed that R4C present no significant similarity to any known phages in the database. Additionally, the predicted ORFs of R4C showed limited sequence identity to those of known phages on both nucleotide and protein levels. The genomic comparison between R4C and R5C (the other published siphophage infecting *D. shibae* DFL12) demonstrated that only 6 homologous genes, 4 of which are annotated as GTA-like genes (showing 31–48% aa identity), are shared between them. To assign phage R4C to one of the previously established phage clusters, phylogenetic analyses based on the major capsid protein and TerL genes were performed (Fig. 4). These two genes have been previously used as markers to study evolutionary relationships as they are considered universally conserved amongst phages. On both phylogenetic trees, R4C was most closely related to three *Rhodobacter* phages belonging to the genus *Cronusvirus* [27] than to other known phages. A systematic sequence comparison between R4C and the *Rhodobacter* phage RcCronus, the representative isolate of the genus *Cronusvirus*, was made with a BLAST genome alignment. The result showed that R4C shares a similar overall genomic organization with RcCronus (Fig. 5). In both genomes, the left-most part is devoted to DNA packaging. Downstream, there is a region putatively related to the virion structure. Following the GTA-like operon, the right-hand region, predicted to be responsible for DNA manipulation and regulation, starts with a gene encoding ribonuclease. In general, R4C and RcCronus share a degree of commonality in the left module, with similarities ranging from 33 to 62% at the amino-acid level. A dramatic departure is seen in the segment downstream from the gene that encodes the transcriptional regulator (ORF 20 in R4C). Although the functions of this segment are unclear, their general proximity to the integrase gene (ORF 29 in R4C) suggests that at least some of these genes may be associated with the phage–host interaction. In addition, there are several notable differences between R4C and RcCronus. First, an integrase gene is predicted to occur in R4C, but is absent from RcCronus (Fig. 5), suggesting that these two phages may have distinct lytic/lysogenic decision mechanisms. This possibility is supported by the observation that RcCronus contains a lysozyme gene downstream from its terminase genes, whereas there is no corresponding gene in the R4C genome. It is also noteworthy that the putative single-stranded-DNA-binding protein (SSB) predicted for R4C is positioned in the left module, in contrast to RcCronus SSB, which is next to the transcriptional-regulator-related gene. Overall, the conserved genomic arrangement suggests that R4C and the *Cronusvirus* phage share a common ancestor. However, only five pairs of homologous sequences show > 50% amino-acid identity and several crucial genes (e.g. integrase/lysozyme) differ in their presence/absence between the two genomes, suggesting substantial divergence between these two viruses.

### Recruitment of metagenomic data

Based on metagenomic recruitment, homologues of R4C were found in diverse habitats, ranging from coastal waters to the open ocean (Additional file 1: Figure S1, Table 1). The highest recruitment number came from the samples from coastal areas of POV, which is consistent with the isolation environment of R4C. The distribution of recruited reads from the POV and GOS metagenomes showed a similar pattern insofar as the homologues were more prevalent in coastal and intermediate areas than in the open ocean, which seems to mirror the biogeographic pattern of the *Roseobacter* clade.

**Table 1:** Characteristics of R4C ORF homologous reads recruited from different metagenomes

Virome	No. reads	Proportion of ORF homologue (%)	ORF coverage (%)	ORF aa identity (%)
POV-coastal	3,502,573	0.63	39	22–86
POV-intermediate	676,284	0.45	14	21–82
POV-open ocean	1,941,935	0.32	31	25–80
GOS-coastal	3,245,119	0.40	39	21–82
GOS-estuary	321,772	0.48	31	21–59
GOS-open ocean	3,371,631	0.23	33	20–70

*POV* Pacific Ocean Virome, *GOS* Global Ocean Sampling

## Conclusion

In this study, a novel representative of the roseophages was characterized in terms of its microbiological characteristics, genomic organization, phylogenetic relationships, and geographic distribution. Phylogenetic and comparative genomic analyses showed that R4C is a new member of the family *Siphoviridae*. The integrase gene in R4C implies that the phage has a potential lysogenic cycle. Ecologically, a metagenomic analysis showed that the homologues of R4C are more prevalent in coastal areas than in the open ocean. Our comprehensive analysis of this new phage provides insights into the diversity of the tailed phages and the evolutionary relationships between the roseophages and roseobacters. The information provided should also be a useful reference for the identification of the bacterial hosts of phages retrieved from viral metagenomes.

## Supplementary information

**Additional file 1: Table S1.** Bacterial strains used in the host range test and their susceptibility to R4C (+: infected; −: uninfected). **Table S2. **Annotated genes of R4C. **Figure S1.** Presence of homologues of R4C ORFs in various metagenomic databases.

## Data Availability

All data generated or analyzed during this study are included in this published article.

## Abbreviations

CsCl: Cesium chloride
TerL: Large terminase subunit
GOS: Global Ocean Sampling
GTA: Gene transfer agent
NCBI: National Center for Biotechnology Information
ORFs: Open reading frames
PCR: Polymerase chain reaction
PE: Pair-end
PEG: Polyethylene glycol
PFU: Plaque forming unit
POV: Pacific Ocean Virome
SM: Sodium chloridemagnesium sulfate
SSB: Single-stranded-DNA-binding protein
TEM: Transmission electron microscopy
