# Supplementary material for: A newly isolated roseophage represents a distinct member of Siphoviridae family
Source: Virol J. 2019 Nov 6;16:128. doi: 10.1186/s12985-019-1241-6 (PMC6836515; doi:10.1186/s12985-019-1241-6)
Supplement: Supplementary file 1 — Additional file 1: Table S1. Bacterial strains used in the host range test and their susceptibility to R4C (+: infected; −: uninfected). Table S2. Annotated genes of R4C. Figure S1. Presence of homologues of R4C ORFs in various metagenomic databases. [file 12985_2019_1241_MOESM1_ESM.docx]

**Additional file 1**

**Table S1. Bacterial strains used in the host range test and their susceptibility to R4C (+: infected; -: uninfected).**

| **Strain** | **Susceptibility** |
| --- | --- |
| *Dinoroseobacter* sp. DFL12^T^ | + |
| *Dinoroseobacter* sp. JL1447 | - |
| *Roseobacter denitrificans* OCh 114 | - |
| *Hoeflea phototrophica* sp. DFL43 | - |
| *Erythrobacter litoralis* DMS 8509 | - |
| *Erythrobacter longus* DSM 6997 | - |
| *Erythrobacter* sp. JL475 | - |
| *Citromicrobium* sp. WPS32 | - |
| *Citromicrobium* sp. JL2201 | - |
| *Citromicrobium* sp. JL1351 | - |
| *Citromicrobium* sp. JL354 | - |
| *Citromicrobium* sp. JL1363 | - |
| *Silicibacter pomeroyi* DSS3 | - |
| *Roseomonas* sp. JL2290 | - |
| *Roseomonas* sp. JL2293 | - |
| *Roseovarius* sp. JL2434 | - |
| *Paenibacillus* sp. JL1210 | - |
| *Ruegeria* sp. JL126 | - |
| *Sphingobium* sp. JL1088 | - |

**Table S2. Annotated genes of R4C.**

| **ORF No.** | **start** | **stop** | **Strand** | **aa length** | **Putative function** |
| --- | --- | --- | --- | --- | --- |
| 1 | 85 | 717 | + | 211 | terminase small subunit |
| 2 | 710 | 2806 | + | 699 | terminase large subunit |
| 3 | 2810 | 3259 | + | 150 | single-stranded DNA-binding |
| 4 | 3270 | 3806 | + | 179 | head-to-tail joining protein |
| 5 | 3803 | 5458 | + | 552 | portal protein |
| 6 | 5455 | 7344 | + | 630 | major capsid protein |
| 7 | 7344 | 7613 | + | 90 | hypothetical protein |
| 8 | 7613 | 7810 | + | 66 | hypothetical protein |
| 9 | 7803 | 8111 | + | 103 | head-tail joining |
| 10 | 8113 | 8505 | + | 131 | major tail |
| 11 | 8505 | 8852 | + | 116 | tail assembly |
| 12 | 9009 | 10805 | + | 599 | tape measure protein |
| 13 | 10816 | 11460 | + | 215 | GTA ORF12-like |
| 14 | 11457 | 12332 | + | 292 | GTA ORF13-like |
| 15 | 12325 | 12936 | + | 204 | GTA ORF14-like |
| 16 | 12936 | 17279 | + | 1448 | GTA ORF15-like |
| 17 | 17285 | 17977 | + | 231 | ribonuclease III |
| 18 | 17974 | 18396 | + | 141 | hypothetical protein |
| 19 | 18408 | 18617 | + | 70 | hypothetical protein |
| 20 | 18610 | 19740 | + | 377 | transcriptional regulator |
| 21 | 19737 | 20120 | + | 128 | hypothetical protein |
| 22 | 20122 | 20367 | + | 82 | hypothetical protein |
| 23 | 20378 | 20908 | + | 177 | hypothetical protein |
| 24 | 21000 | 21407 | + | 136 | hypothetical protein |
| 25 | 21404 | 21652 | + | 83 | hypothetical protein |
| 26 | 21654 | 21890 | + | 79 | hypothetical protein |
| 27 | 21887 | 21997 | + | 37 | hypothetical protein |
| 28 | 22013 | 22378 | + | 122 | hypothetical protein |
| 29 | 22383 | 23705 | + | 441 | integrase |
| 30 | 23702 | 24043 | + | 114 | hypothetical protein |
| 31 | 24224 | 25246 | + | 341 | hypothetical protein |
| 32 | 25322 | 25579 | + | 86 | hypothetical protein |
| 33 | 25607 | 26029 | + | 141 | hypothetical protein |
| 34 | 26701 | 26117 | − | 195 | hypothetical protein |
| 35 | 26885 | 27157 | + | 91 | hypothetical protein |
| 36 | 27157 | 27429 | + | 91 | hypothetical protein |
| 37 | 27426 | 28706 | + | 427 | DNA methylase |
| 38 | 28718 | 29008 | + | 97 | hypothetical protein |
| 39 | 29005 | 29901 | + | 299 | helix-turn-helix domain-containing protein |
| 40 | 29898 | 30218 | + | 107 | hypothetical protein |
| 41 | 30215 | 30469 | + | 85 | hypothetical protein |
| 42 | 30466 | 31062 | + | 199 | phosphohydrolase |
| 43 | 31062 | 32240 | + | 393 | DNA primase |
| 44 | 32242 | 32433 | + | 64 | hypothetical protein |
| 45 | 32426 | 32815 | + | 130 | hypothetical protein |
| 46 | 32827 | 34479 | + | 551 | DNA primase/helicase |
| 47 | 34479 | 34664 | + | 62 | hypothetical protein |
| 48 | 34847 | 35509 | + | 221 | hypothetical protein |
| 49 | 35640 | 36245 | + | 202 | minor tail |

**
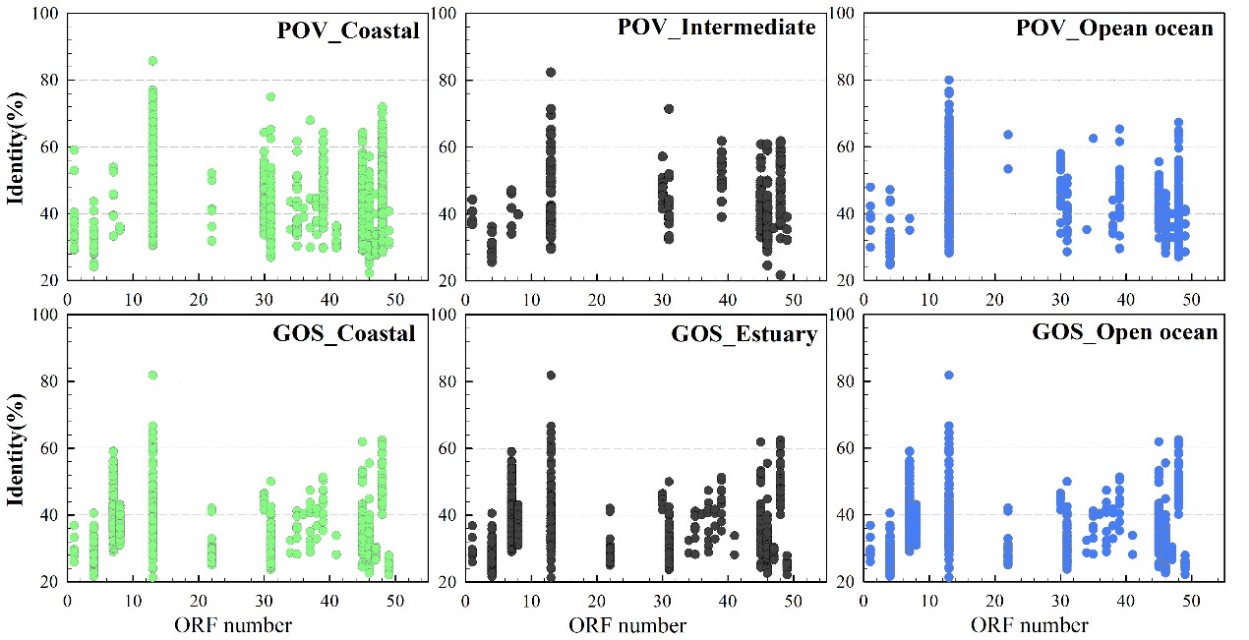
**

**Figure S1. Presence of homologues of R4C ORFs in various metagenomic databases.**
